# Assisted Reductive Amination for Quantitation of Tryptophan, 5-Hydroxytryptophan, and Serotonin by Ultraperformance Liquid Chromatography Coupled with Tandem Mass Spectrometry

**DOI:** 10.3390/molecules28124580

**Published:** 2023-06-06

**Authors:** Shih-Shin Liang, Po-Tsun Shen, Yu-Qing Liang, Yi-Wen Ke, Chieh-Wen Cheng, Yi-Reng Lin

**Affiliations:** 1 Department of Biotechnology, College of Life Science, Kaohsiung Medical University, Kaohsiung 80708, Taiwan; monged180@gmail.com (Y.-Q.L.); jjj52021@yahoo.com.tw (Y.-W.K.); 2 Institute of Biomedical Science, College of Medicine, National Sun Yat-sen University, Kaohsiung 80424, Taiwan; 3 Research Center for Precision Environmental Medicine, Kaohsiung Medical University, Kaohsiung 80708, Taiwan; 4 Department of Medical Research, Kaohsiung Medical University Hospital, Kaohsiung 80708, Taiwan; 5 Protein Chemistry Core Laboratory, Core Instrument Center, National Health Research Institutes, Miaoli 35053, Taiwan; jasonpika@gmail.com; 6 Bachelor Program in Industrial Technology, College of Future, National Yunlin University of Science and Technology, Yunlin 64002, Taiwan; jwencmc@yuntech.edu.tw; 7 Department of Biotechnology, School of Environment and Life Sciences, Fooyin University, Kaohsiung 83102, Taiwan

**Keywords:** reductive amination, serotonin, 5-hydroxytryptophan, tryptophan, multiple reaction monitoring

## Abstract

Herein, we used isotopic formaldehyde and sodium cyanoborohydride via reductive amination to label two methyl groups on primary amine to arrange the standards (*h*_2_-formaldehyde-modified) and internal standards (ISs, *d*_2_-formaldehyde-modified) of tryptophan and its metabolites, such as serotonin (5-hydroxytryptamine) and 5-hydroxytryptophan. These derivatized reactions with a high yield are very satisfactory for manufacturing standards and ISs. This strategy will generate one or two methyl groups on amine to create different mass unit shifts with 14 vs. 16 or 28 vs. 32 in individual compounds for biomolecules with amine groups. In other words, multiples of two mass units shift are created using this derivatized method with isotopic formaldehyde. Serotonin, 5-hydroxytryptophan, and tryptophan were used as examples to demonstrate isotopic formaldehyde-generating standards and ISs. *h*_2_-formaldehyde-modified serotonin, 5-hydroxytryptophan, and tryptophan are standards to construct calibration curves, and *d*_2_-formaldehyde-modified analogs such as ISs spike into samples to normalize the signal of each detection. We utilized multiple reaction monitoring modes and triple quadrupole mass spectrometry to demonstrate the derivatized method suitable for these three nervous biomolecules. The derivatized method demonstrated a linearity range of the coefficient of determinations between 0.9938 to 0.9969. The limits of detection and quantification ranged from 1.39 to 15.36 ng/mL.

## 1. Introduction

Neurotransmitters are chemical molecules that are released from axon terminals with neural impulses [1]. These chemical molecules include glutamate [2], 5-hydroxytryptamine (serotonin, 5-HT) [3], dopamine, noradrenaline [4], epinephrine [5], adenosine [6], etc. Neurotransmitters have multiple functions in the nervous system, i.e., dopamine plays an important role in regulating neuroendocrine functions, motivation, emotion, and locomotor activity [7], and serotonin affects human beings’ mood via maintaining high concentrations in the blood [8].

In the syntheses of neurotransmitters from neural precursors, tryptophan (TRP), an original component, is catalyzed using tryptophan hydroxylase (TPH) to generate 5-hydroxytryptophan (5-HTP) [9], and serotonin is generated from 5-HTP, which is catalyzed by aromatic L-amino acid decarboxylase (AAAD) [10]. The other important bioactive metabolite, melatonin, is produced by serotonin reacted with N-acetyl transferase and 5-hydroxyindole-*O*-methyltransferase [11]. The other neurotransmitter, dopamine, is used by tyrosine to be catalyzed by AAAD [12,13]. TRP is an essential amino acid for protein synthesis and has three pathways in TRP metabolism, including the serotonin-melatonin pathway [10,14], indole-3-acetic acid pathway [10], and kynurenine pathway [15,16,17]. An imbalance in the levels of TRP and its metabolites will cause neuronal damage and impair physiological and neurological functions [18,19]. The TRP metabolite of serotonin is also an important neurotransmitter, and regulates numerous human behaviors, emotion, appetite, memory and learning [20]. Therefore, all the metabolites in the pathways reflect behavioral and physiological significances.

Nowadays, metabolomics demonstrate a novel vision and the possibility of screening metabolites with powerful high-throughput instruments [21,22,23,24]. Among the separated metabolomics system, high-performance liquid chromatography coupled with tandem mass spectrometry (HPLC–MS/MS) is widely used in many research fields [25,26,27,28]. Tryptophan metabolic pathways produce important bioactive substances, and therefore, TRP and its metabolites are important in cell metabolism. Therefore, we selected the serotonin pathway as an example, with TRP, 5-HTP, and serotonin as target molecules, to develop a high-performance liquid chromatography–tandem mass spectroscopy quantitative platform. However, in this technique, each sample detection needed internal standards (ISs) to normalize the intensity of individual samples [15]. In the previous literature, the quantitation of TRP, kynurenine, kynurenic acid and serotonin occurred via reverse-phase column (TSK-Gel ODS-80 Ts) coupled with UV-Vis spectroscopy, fluorescence, and mass spectrometry [29]. The results showed that TRP, kynurenine, kynurenic acid and serotonin had fluorescence and UV absorbance functional groups without any dyes labeling. The other method involves the use of capillary electrophoresis coupled with mass spectrometry to determine serotonin, TRP, and 5-HTP in human plasma. The results demonstrated that the concentration levels were below 100 ppb (sub-μM) [30]. The HPLC/MS/MS instrument was set up with two mass analyzers to select *m*/*z* of the precursor ion at the first mass analyzer and to select *m*/*z* of the product ions at the secondary mass analyzer after fragmentation. This detection mode of tandem MS is named multiple-reaction monitoring (MRM) and has advantages, such as improvement of sensitivity and selectivity. In rat brain samples, MRM detection mode was utilized to monitor serotonin and TRP [31,32]. However, in traditional HPLC/MS analyses, a better analytical strategy is the use of isotope internal standards such as ISs to normalize the intensity of signals in every sample.

Previously, we developed a derivatized method to generate ISs beneficial for HPLC–MS/MS analyses [33,34,35,36,37,38,39]. Herein, we used isotopic formaldehyde and sodium cyanoborohydride to generate two methyl groups on the amine functional group of TRP, 5-HTP, and serotonin. This modification, with cheap and easily obtained materials, showed signal enhancement and helped target molecules to be monitored [40]. Eventually, we establish the derivatized method by estimating calibration curves, the limit of detection (LOD), the limit of quantification (LOQ), intra-day precision, and inter-day precision.

## 2. Results

### 2.1. Reductive Amination of Tryptophan and Its Metabolites

The TRP pathway shows that TRP can be metabolized using TPH to be transformed into 5-HTP and serotonin, an important neurotransmitter. The conventional method to determine the concentrations of TRP, 5-HTP, and serotonin uses HPLC–MS/MS, and ISs are needed for each sample detection. In general, isotopic analogs, such as deuterium replacing hydrogen, are used to spike the samples during pretreatment to recognize the individual signals of each sample [15,41].

**Figure 1:**
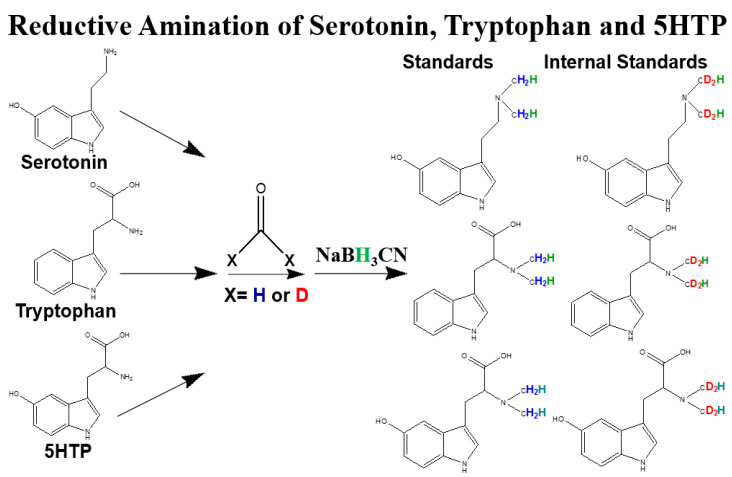
Schematic diagram of the reductive amination for serotonin, tryptophan, and 5-HTP. The modification used isotopic formaldehyde and reduction with sodium cyanoborohydride (NaBH_3_CN).

Herein, we developed a modified method to generate TRP, 5-HTP, and serotonin with *h*_2_-formaldehyde and *d*_2_-formaldehyde to generate *h*_2_-formaldehyde-modified TRP, 5-HTP, and serotonin (as standards) and to react with *d*_2_-formaldehyde-modified TRP, 5-HTP, and serotonin (as ISs). Figure 1 shows the schematic diagram of this derivatized method (reductive amination), in which isotopic formaldehyde reacts and reduces using NaBH_3_CN.

### 2.2. MS/MS Fragmentation and MRM Transition Setting

**Figure 2:**
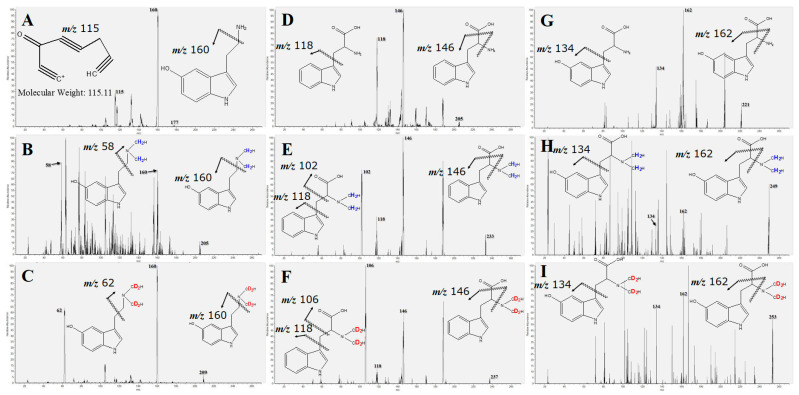
The mass spectra of fragmented ions to set the multiple-reaction-monitoring (MRM) methods, the ion-pairs of mass-to-charge ratios (*m*/*z*) of the precursor, and product fragments. (**A**) Unmodified serotonin (*m*/*z* = 177) and its product ions at *m*/*z* = 115 and 160, (**B**) *h*_2_-derivatized serotonin (*m*/*z* = 205) and its product ions at *m*/*z* = 58 and 160, (**C**) *d*_2_-derivatized serotonin (*m*/*z* = 209) and its product ions at *m*/*z* = 62 and 160, (**D**) unmodified tryptophan (TRP) (*m*/*z* = 205) and its product ions at *m*/*z* = 118 and 146, (**E**) *h*_2_-derivatized TRP (*m*/*z* = 233) and its product ions at *m*/*z* = 102, 118, and 146, (**F**) *d*_2_-derivatized TRP (*m*/*z* = 237) and its product ions at *m*/*z* = 106, 118, and 146, (**G**) unmodified 5-HTP (*m*/*z* = 221) and its product ions at *m*/*z* = 134 and 162, (**H**) *h*_2_-derivatized 5-HTP (*m*/*z* = 249) and its product ions at *m*/*z* = 134 and 162, and (**I**) *d*_2_-derivatized 5-HTP (*m*/*z* = 253) and its product ions at *m*/*z* = 134 and 162.

The multiple-reaction-monitoring (MRM) transitions of TRP and its metabolites were determined using MS/MS fragmentation. All unmodified and isotopic formaldehyde-modified compounds were determined using tandem MS. An individual compound was infused into tandem MS and fragmented by colliding gas. The MS/MS spectrum of unmodified serotonin (Figure 2A) showed a precursor ion at mass-to-charge ratio (*m*/*z*) = 177, and its fragments showed *m*/*z* values of 160 and 115 (product ions, the structure of *m*/*z* 115 refers to the website of The Human Metabolome Database, https://hmdb.ca/metabolites/HMDB0000259 (accessed on 30 May 2023)). Serotonin was modified using reductive amination via two methyl groups, and the molecular weight was transformed into 204 (*h*_2_-modified serotonin, *m*/*z* 205) and 208 (*d*_2_-modified serotonin, *m*/*z* 209). As shown in Figure 2B, 205 > 58 and 205 > 160 were fragmented from the precursor ions of *h*_2_-modified serotonin. Meanwhile, as shown in Figure 2C, 209 > 62 and 209 > 160 were fragmented from the precursor ions of *d*_2_-modified serotonin. The difference between 205 > 58 and 209 > 62 supported that reductive amination achieved isotopic modification. Similarly, unmodified TRP with an *m*/*z* value of 205 was fragmented to generate product ions at *m*/*z* = 118 and 146 (Figure 2D). The fragmentations of *h*_2_-modified TRP and *d*_2_-modified TRP were attributed to 233 > 102, 233 > 118 and 233 > 146 (Figure 2E), and 237 > 106, 237 > 118 and 237 > 146 (Figure 2F), respectively. Finally, the MRM transitions setting from unmodified 5-HTP was assigned according to 221 > 134 and 221 > 162 (Figure 2G). *h*_2_-modified 5-HTP exhibited the MRM transitions of 249 > 134 and 249 > 162 (Figure 2H); meanwhile, *d*_2_-modified 5-HTP exhibited the MRM transitions of 253 > 134 and 253 > 162 (Figure 2I). The MRM transitions of serotonin, tryptophan, and 5-HTP and their modified analogs are listed in Table 1, including the molecular formula, molecular weight, LOD, LOQ, and coefficient of determination (*R*^2^).

### 2.3. Signal Enhancements of Modified Serotonin, TRP, and 5-HTP

**Figure 3:**
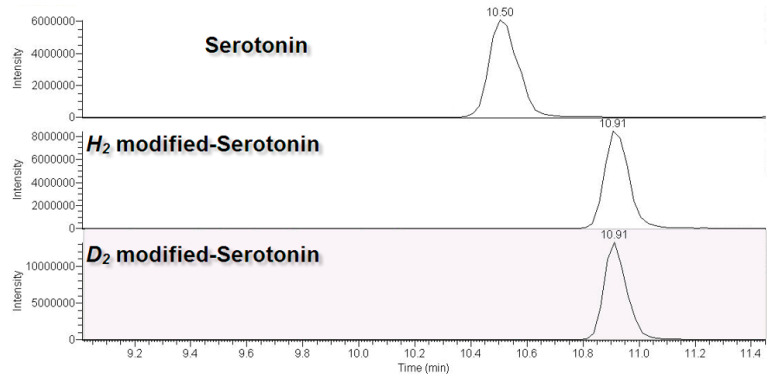
Signal enhancement of serotonin via reductive amination and a comparison of the peak areas of the unmodified, *h*_2_-modified, and *d*_2_-modified analogs: unmodified serotonin (*m*/*z* = 177, retention time [rt] = 10.50 min, *y*-axis intensity = 6.0 × 10^6^), *h*_2_-modified serotonin (*m*/*z* = 205, rt = 10.91 min, *y*-axis intensity = 8.2 × 10^6^), and *d*_2_-modified serotonin (*m*/*z* = 209, rt = 10.91 min, *y*-axis intensity = 1.6 × 10^7^).

The peak areas of the MS spectra between the unmodified and modified isotopic formaldehyde were compared to determine the signal enhancement in modified samples. We selected serotonin as an example, as shown in Figure 3. Identical amounts of unmodified, *h*_2_-derivatized, and *d*_2_-derivatized serotonin were mixed to extract the MS/MS spectra of the unmodified serotonin (MRM transitions: *m*/*z* = 221 > 162 and 221 > 134, retention time [rt] = 10.50 min, absolute intensity = 6.0 × 10^6^), *h*_2_-derivatized serotonin (MRM transitions: *m*/*z* = 249 > 162 and 249 > 134, rt = 10.91 min, absolute intensity = 8.2 × 10^6^), and *d*_2_-derivatized serotonin (MRM transitions: *m*/*z* = 253 > 162 and 253 > 134, rt = 10.91 min, absolute intensity = 1.6 × 10^7^). According to the same procedures, we compared the integrated areas (average value, *n* = 4) of the unmodified and *d*_2_-derivatized analogs of serotonin, TRP, and 5-HTP. The datasets showed a signal enhancement of 1.19-fold (serotonin), 18.87-fold (TRP), and 2.40-fold (5-HTP) upon derivatization. The integrated and statistical results, including the average integrated area, relative standard deviation, and signal enhancement, are shown in Table 2. We have previously discussed the factor that enhances signals via reductive amination [40]. We estimated that the factor is the amine group showing a higher electronic density after modification with two methyl groups, causing the lone pair in the amine group to show a more basic quality.

### 2.4. Method Validation

To estimate the applicability of this derivatized method for serotonin, TRP, and 5-HTP, we used the modified three compounds to generate *h*_2_-formaldehyde-derivatized analogs to construct calibration curves and calculate *R*^2^, LOD, and LOQ. The *h*_2_-derivatized serotonin, TRP, and 5-HTP standards required to construct the calibration curve were prepared using 180 μL at concentrations of 1.0, 2.0, 5.0, 10.0, 20.0, 50.0, 100, 200, 500, and 1000 ng/mL (ppb). Meanwhile, the *d*_2_-derivatized ISs were spiked with an identical 20 μL of 2.0 ppm *d*_2_-modified serotonin, TRP, and 5-HTP. Each calibration curve was extracted from the normalized MS/MS spectra of the *h*_2_-modified serotonin, TRP, and 5-HTP using their *d*_2_-modified analogs. The *R*^2^ of the calibration curves ranged from 0.9938 to 0.9969 for *h*_2_-modified analogs. The statistical data are listed in Table 1. The validation parameters of the LODs and LOQs for the modified analogs were 2.35 and 14.54 ng/mL for serotonin, 1.96 and 4.36 ng/mL for tryptophan, and 1.39 and 15.36 ng/mL for 5-HTP, respectively. A concentration of 1 ng/mL was injected as a low-concentration specimen to estimate the LOD and LOQ. The data of the intra-day and inter-day concentrations are shown in Table 3.

The other validations to assess isotopic *d*_2_-modified analogs are compatible with serotonin, TRP, and 5-HTP quantitative analysis. We spiked different ratios of isotopic amount, including 20:1, 10:1, 5:1, 1:1, 1:5, 1:10, and 1:20. We selected serotonin as an example to demonstrate the effect of different *h*_2_:*d*_2_ ratios in detecting serotonin, while showing the corresponding ratios of signal area integrations.

**Figure 4:**
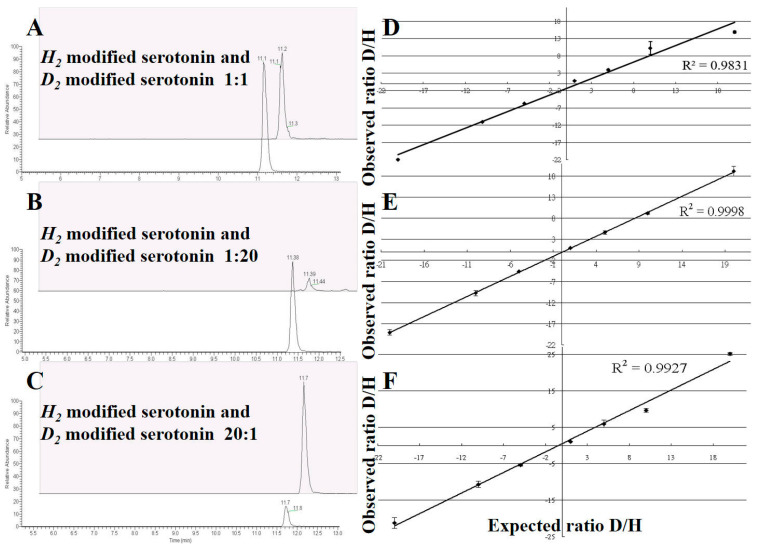
Demonstration of the applicability of serotonin, tryptophan, and 5-HTP derivatization with different ratios of *h*_2_-modified and *d*_2_-modified analogs. (**A**) Serotonin analogs blended with *h*_2_-modified vs. *d*_2_-modified at 1:1, (**B**) serotonin analogs blended with *h*_2_-modified vs. *d*_2_-modified at 1:20, (**C**) serotonin analogs blended with *h*_2_-modified vs. *d*_2_-modified at 20:1, (**D**) serotonin analogs blended with different ratios of *h*_2_:*d*_2_ analogs, including 20:1 (20), 10:1 (10), 5:1 (5), 1:1 (1), 1:5 (−5), 1:10 (−10), and 1:20 (−20), and illustrating the expected ratios vs. observed ratios. The coefficient of determination is 0.9831. (**E**) Tryptophan analogs illustrated the expected ratios vs. observed ratios with a coefficient of determination at 0.9998. (**F**) 5-HTP analogs illustrated the expected ratios vs. observed ratios with a coefficient of determination at 0.9927.

Figure 4A–C show the ratios of *h*_2_-modified to *d*_2_-modified serotonin, including 1:1, 1:20, and 20:1. The dynamic linear range for the quantitative analyses of the serotonin, TRP, and 5-HTP estimated an *R*^2^ of 0.9831 (Figure 4D), 0.9998 (Figure 4E), and 0.9927 (Figure 4F), respectively.

## 3. Materials and Methods

### 3.1. Materials

Serotonin (hydrochloride form), sodium cyanoborohydride (NaBH_3_CN), sodium acetate, and tryptophan were obtained from Sigma–Aldrich (St. Louis, MO, USA). The LC–MS grade solvents, including acetonitrile (MeCN), methanol (MeOH), and 0.22 μm PTFE filters, were bought from Merck (Seelze, Germany). Sodium hydroxide and hydrochloric acid were purchased from J.T. Baker (Phillipsburg, NJ, USA) to adjust the buffer pH. Formaldehyde-*D*_2_ solution (20% solution in D_2_O) was purchased from Isotec Corp. (Miamisburg, OH, USA), and formaldehyde-*H*_2_ solution (36.5–38% in H_2_O), 5-HTP and formic acid (FA, 98–100%) were purchased from Sigma (St. Louis, MO, USA). Deionized H_2_O with a resistance of 18.2 MΩ was obtained using a Millipore water system.

### 3.2. Instrumentation

The instruments for separation and detection, including ultrahigh-performance liquid chromatography coupled with a tandem mass spectrometer (UHPLC–MS/MS, Thermo Fisher Scientific Inc., Waltham, MA, USA), were equipped with a Thermo Finnigan Acella 1250 UHPLC system (Thermo Fisher Scientific Inc., Waltham, MA, USA). The MS system was a triple quadrupole MS (Thermo Fisher Scientific Inc., Waltham, MA, USA). The inlet system of a microelectrospray ionization (ESI, Thermo Fisher Scientific Inc.) ion source was set in the positive ion mode with an applied voltage of 3000 V, and the vaporizing and capillary temperatures were set at 300 °C and 350 °C, respectively. The auxiliary gas and sheath gas pressures were set to 10 units and 30 units, respectively. The collision energy was adjusted to 25 V with high-quality argon gas. The MS survey scan mode of MRM was employed to select the precursor and product ions for fragmentation with the MRM transitions setting, as shown in Table 1. The separated system used a 10-μL sample injected into a loop using an Acella 1250 autosampler and separated by a Shiseido CAPCELL PAK MG II C18 column (i.d. 1.5 mm × 150 mm, 3 μm, Tokyo, Japan). The flow rate of UHPLC was set at 200 μL/min with mobile phases of 0.1% FA in water (A) and 0.1% FA in 100% MeCN (B). The linear gradient was set as follows: 2% (B) for 1 min; 2–40% (B) for 5 min; 40–98% (B) for 3 min, followed by holding at 98% (B) for 2 min. The software to control and adjust the UHPLC and MS instrument systems was Xcalibur (version 2.2, Thermo Finnigan Inc., San Jose, CA, USA), and Xcalibur was also utilized to acquire MS data. The statistics analyses used the Xcalibur Thermo LCquan software (version 2.7, Thermo Finnigan Inc., San Jose, CA, USA).

### 3.3. Preparation of Derivatized Standards and Internal Standards

Serotonin, 5-HTP, and TRP were weighed and dissolved in dimethyl sulfoxide at 100 mg/mL (100,000 ppm) and stored in a refrigerator at −80 °C as stock solutions. A mixture of serotonin, 5-HTP, and TRP was dissolved, and the concentration was adjusted to 100 ppm with 50% MeCN (*v*/*v*) solution. The reductive amination procedures were used in a mixture of 20 μL of 100 ppm blended with 160 μL of 50 mM sodium acetate buffer (pH 5.6) and 10 μL of 4% *h*_2_-formaldehyde to generate standards. To derivatize for the generation of serotonin, 5-HTP, and TRP ISs, *h*_2_-formaldehyde was replaced by 4% *h*_2_-formaldehyde. After 5 min, 10 μL of NaBH_3_CN was spiked into each tube to reduce the derivatized serotonin, 5-HTP, and TRP. A diagram of reductive amination is illustrated in Figure 1.

### 3.4. Development of the Detection Method for Serotonin, 5-Hydroxytryptophan, and Tryptophan

We used MRM for the detection of *h*_2_-formaldehyde- and *d*_2_-formaldehyde-modified serotonin, 5-HTP, and TRP analogs. Individual serotonin, 5-HTP, and TRP analogs, including unmodified, *h*_2_-formaldehyde-generated, and *d*_2_-formaldehyde-generated compounds, were infused into the MS instrument using a syringe and fragmented using argon gas in an MS collision chamber. The ions, including the selected precursor and fragmented product ions, of the unmodified and modified analogs are shown in Figure 2. The fragmentation of the MS spectra was extracted to arrange the MRM transitions listed in Table 1.

### 3.5. Evulation of the Derivitized Method

The calibration curves for serotonin, 5-HTP, and TRP were constructed using the concentrations of *h*_2_-modified analogs: 1, 2, 5, 10, 20, 50, 100, 200, 500, and 1000 ng/mL (ppb). The serotonin, 5-HTP, and TRP calibration curves also determined the intra-day and inter-day LODs and LOQs. In each sample solution (180 μL), an equal volume (20 μL, 2 ppm) of *d*_2_-modified serotonin, 5-HTP, and TRP analogs was added as ISs to each Eppendorf tube. The statistical data for the intra-day and inter-day reproducibility of serotonin, 5-HTP, and TRP were calculated for triplicate experiments (*n* = 3), respectively. The LOD and LOQ were determined using low-concentration samples (1 ng/mL of serotonin, TRP, and 5-HTP samples, [*n* = 6]). To validate this modified method for serotonin, 5-HTP, and TRP, we used *h*_2_-formaldehyde and *d*_2_-formaldehyde products mixed at different ratios of 20:1, 10:1, 5:1, 1:1, 1:5, 1:10, and 1:20 to demonstrate the linear range of the quantitative analysis via a reductive amination method. The results are shown in Figure 4. Figure 4A–C show the ratios of 1:1 (1), 1:20 (20-fold), and 20:1 (1/20, −20), respectively.

## 4. Conclusions

A convenient and affordable derivatized method was developed, easily compatible with HPLC–MS/MS. The derivatized method used the isotopic formaldehyde-modified serotonin, 5-HTP, and TRP for quantification. The derivatized method showed labor-saving qualities, a high yield, and, most importantly, promoted signal enhancement. We demonstrated three orders of dynamic linear ranges, and the individual coefficients of determination were 0.9938 and 0.9969. Intra-day and inter-day data indicated a stable reproducibility, and the LOD and LOQ ranged between 1.39 and 15.36 ng/mL.

Therefore, we considered that this reductive amination-based derivatized method could screen the serotonin pathway, including TRP and its metabolites, serotonin, and 5-HTP.

## Figures and Tables

**Table 1:** Modified and unmodified molecules of serotonin, tryptophan and 5-HTP with molecular formula, molecular weight, MRM transitions with *m/z* of precursor ion and *m/z* of product ions, LOD and LOQ parameters are listed.

Name	Molecular Formula	Molecular Weight (Da)	MRM Transitions		LOD ^#^ (ng/mL)	LOQ ^#^ (ng/mL)	Coefficient of Determination (R^2^)
			Precursor Ion	Product Ions			
Serotonin	C_10_H_12_N_2_O	176.22	177	115, 160	---	---	---
*h*_2_-modified serotonin	C_12_H_16_N_2_O	204.27	205	58, 160	2.35	14.54	0.9969
*d*_2_-modified serotonin	C_12_H_12_D_4_N_2_O	208.29	209	62, 160	---	---	---
Tryptophan	C_11_H_12_N_2_O_2_	204.23	205	118, 146	---	---	---
*h*_2_-modified tryptophan	C_13_H_16_N_2_O_2_	232.28	233	102, 118, 146	1.96	4.36	0.9959
*d*_2_-modified tryptophan	C_13_H_12_D_4_N_2_O_2_	236.30	237	106, 118, 146	---	---	---
5-HTP	C_11_H_12_N_2_O_3_	220.22	221	134, 162	---	---	---
*h*_2_-modified 5-HTP	C_13_H_16_N_2_O_3_	248.28	249	134, 162	1.39	15.36	0.9938
*d*_2_-modified 5-HTP	C_13_H_12_D_4_N_2_O_3_	252.30	253	134, 162	---	---	---

^#^ Limits of detection (LOD) and limits of quantification (LOQ) values were ascertained following a statistical method, LOD: average minimum recognizable analytical signal of low-concentration samples + 3 × standard deviation of analytical signal of low-concentration samples; LOQ: average analytical signal of low-concentration samples + 10 × standard deviation of analytical signal of low-concentration samples (*n* ≥ 6).

**Table 2:** The signal-enhancement statistical data of modified and unmodified serotonin, tryptophan and 5-HTP in MRM method.

	MRM/Average Peak Area (*n* = 4)				
	Unmodified	RSD (%)	*d*_2_-Modified	RSD (%)	Enhancement
Serotonin	55,030,586	4.2	65,536,481	4.5	1.19-fold
Tryptophan	6,765,226	3.8	127,646,745	1.2	18.87-fold
5-HTP	2,193,503	6.3	5,257,500	2.4	2.40-fold

**Table 3:** Statistical intra-day and inter-day data for serotonin, tryptophan and 5-HTP analogs (*n* = 3).

	Serotonin		Tryptophan		5-HTP	
Concentration (ng/mL)	Intra-Day	Inter-Day	Intra-Day	Inter-Day	Intra-Day	Inter-Day
500	3.5–6.9%	4.0%	1.2–3.8%	6.4%	0.6–6.1%	6.7%
100	2.2–6.4%	3.0%	1.6–8.6%	7.9%	1.7–9.7%	5.7%
50	3.4–3.9%	9.7%	2.0–10.8%	3.7%	4.1–16.3%	6.1%

## Data Availability

We inform that all the published data were obtained and involved in this research.
